# Quantifying the Turnover of Transcriptional Subclasses of HIV-1-Infected Cells

**DOI:** 10.1371/journal.pcbi.1003871

**Published:** 2014-10-23

**Authors:** Christian L. Althaus, Beda Joos, Alan S. Perelson, Huldrych F. Günthard

**Affiliations:** 1 Institute of Social and Preventive Medicine (ISPM), University of Bern, Bern, Switzerland; 2 Division of Infectious Diseases and Hospital Epidemiology, University Hospital Zürich, University of Zürich, Zürich, Switzerland; 3 Theoretical Division, Los Alamos National Laboratory, Los Alamos, New Mexico, United States of America; Imperial College London, United Kingdom

## Abstract

HIV-1-infected cells in peripheral blood can be grouped into different transcriptional subclasses. Quantifying the turnover of these cellular subclasses can provide important insights into the viral life cycle and the generation and maintenance of latently infected cells. We used previously published data from five patients chronically infected with HIV-1 that initiated combination antiretroviral therapy (cART). Patient-matched PCR for unspliced and multiply spliced viral RNAs combined with limiting dilution analysis provided measurements of transcriptional profiles at the single cell level. Furthermore, measurement of intracellular transcripts and extracellular virion-enclosed HIV-1 RNA allowed us to distinguish productive from non-productive cells. We developed a mathematical model describing the dynamics of plasma virus and the transcriptional subclasses of HIV-1-infected cells. Fitting the model to the data allowed us to better understand the phenotype of different transcriptional subclasses and their contribution to the overall turnover of HIV-1 before and during cART. The average number of virus-producing cells in peripheral blood is small during chronic infection. We find that a substantial fraction of cells can become defectively infected. Assuming that the infection is homogenous throughout the body, we estimate an average *in vivo* viral burst size on the order of 10^4^ virions per cell. Our study provides novel quantitative insights into the turnover and development of different subclasses of HIV-1-infected cells, and indicates that cells containing solely unspliced viral RNA are a good marker for viral latency. The model illustrates how the pool of latently infected cells becomes rapidly established during the first months of acute infection and continues to increase slowly during the first years of chronic infection. Having a detailed understanding of this process will be useful for the evaluation of viral eradication strategies that aim to deplete the latent reservoir of HIV-1.

## Author Summary

Gaining a quantitative understanding of the development and turnover of different HIV-1-infected subpopulations of cells is crucial to improve the outcome of patients on combination antiretroviral therapy (cART). The population of latently infected cells is of particular interest as they represent the major barrier to a cure of HIV-1 infection. We developed a mathematical model that describes the dynamics of different transcriptionally active subclasses of HIV-1-infected cells and the viral load in peripheral blood. The model was fitted to previously published data from five chronically HIV-1-infected patients starting cART. This allowed us to estimate critical parameters of the within-host dynamics of HIV-1, such as the the number of virions produced by a single infected cell. The model further allowed investigation of HIV-1 dynamics during the acute phase. Computer simulations illustrate that latently infected cells become rapidly established during the first months of acute infection and continue to increase slowly during the first years of chronic infection. This illustrates the opportunity for strategies that aim to eradicate the virus during early cART as the pool of HIV-1 infected cells is substantially smaller during acute infection than during chronic infection.

## Introduction

High levels of cell-associated HIV-1 RNA can be observed in peripheral blood of patients with undetectable plasma viremia during combination antiretroviral therapy (cART) [1]–[4]. The various HIV-1 RNA and DNA species that are present during the viral life cycle can serve as biomarkers for basal transcription in viral reservoirs with different properties [5], [6]. Gaining a quantitative understanding of the development and turnover of HIV-1-infected subpopulations and viral latency is of particular interest in light of recent efforts in viral eradication strategies [7]–[10].

Highly sensitive assays for HIV-1 plasma RNA in patients on cART usually provide bulk measurements of viral activity and cannot distinguish between different infected subpopulations [11]. In contrast, the study by Fischer et al. [12] combined highly sensitive PCR assays for unspliced (UsRNA) and multiply spliced (MsRNA-tatrev and MsRNA-nef) HIV-1 RNA species with limiting dilution endpoint analysis of peripheral blood mononuclear cells (PBMCs). In addition to intracellular RNA transcripts, extracellular virion-enclosed HIV-1 RNA that provides a marker for cells releasing virus particles was also measured. The study identified four distinct viral transcriptional classes: two overlapping cell classes of high viral transcriptional activity, representative of a virus producing phenotype; and two cell classes that express HIV-1 RNA at low and intermediate levels that match definitions of viral latency [12], [13].

**Figure 1:**
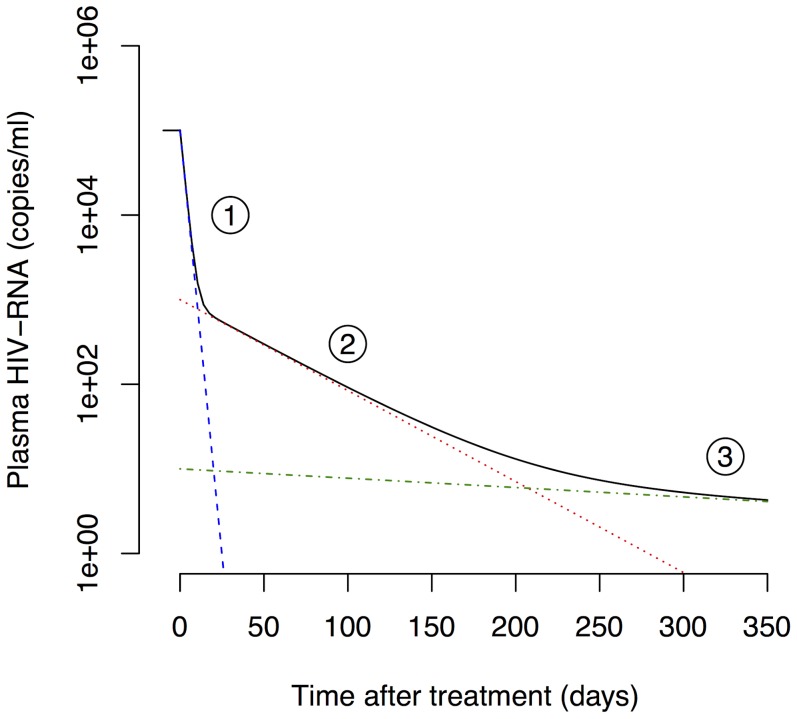
Three-phase decay of HIV-1 after start of cART. The black line shows a typical decay profile of plasma viral load during the first year of cART. Typical half-lives of the first (blue dashed line), second (red dotted line) and third phase (green dash-dotted line) are 1.5 days, 4 weeks and 39 weeks, respectively [22].

Analyzing the decay kinetics of plasma viral load in HIV-1-infected patients on cART using mathematical models has resulted in a detailed understanding of viral replication dynamics *in vivo* [14]–[16]. The plasma viral load typically exhibits three exponential phases during the first year after start of cART (Figure 1). Due to the rapid turnover of free virus in blood [17], the viral decay phases are thought to reflect the contribution of different HIV-1-infected cell populations on viral production. The first phase with a half-life of 1 to 2 days is attributed to the loss of activated, virus-producing cells [18], [19]. The second phase exhibits a half-life of 1 to 4 weeks and is considered to reflect the loss of so-called persistently infected cells with a lower state of activation [20], [21]. The third phase decay has a long half-live of 39 weeks suggesting that latently infected cells are a primary candidate for this cellular compartment [22], [23], although slow release of virus from the follicular dendritic cell network is another possibility [24]. Although not shown in Figure 1, in many patients, after the third phase a final low steady state level of plasma viremia is attained, that has been called a fourth phase [22]. This phase has also been attributed to release of virus from activated latently infected cells [22]. Other mathematical models have been developed that stratify the infected cells into additional subpopulations such as non-productively infected cells during the intracellular eclipse phase [25] and defectively infected cells [26]. Nevertheless, most studies to date are focused on the analysis of viral load and only indirectly allow inferring the kinetics of cellular subpopulations. Few studies have attempted to characterize the concentration of virus and several infected subpopulations based on data simultaneously [26]. Fitting mathematical models to multiple quantities of viral replication would result in refined parameter estimates for describing the generation and maintenance of latently infected cells.

In this study, we developed a mathematical model that describes the dynamics of different transcriptionally active subclasses of HIV-1-infected cells and the viral load in peripheral blood. The model was fitted to previously published data from five chronically HIV-1-infected patients starting cART [12]. This allowed us to estimate critical parameters of the within-host dynamics of HIV-1 and the turnover of various subpopulations of infected cells. Finally, we simulated the development of the latently infected cell pool during acute infection, providing useful information for viral eradication strategies.

## Results

**Figure 2:**
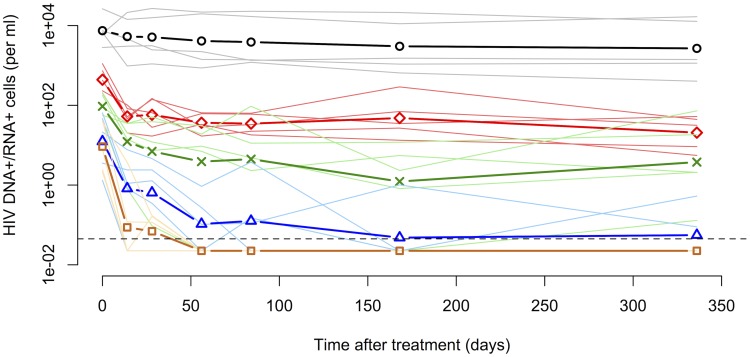
Decay kinetics of subclasses of HIV-1-infected cells during cART. The five subclasses of PBMCs are: DNA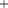 (containing HIV-1 DNA, black circles), Low (containing solely HIV-1 UsRNA, red diamonds), Mid (containing only HIV-1 MsRNA-tatrev or MsRNA-nef, green crosses), High (containing elevated levels of both HIV-1 MsRNA-tatrev and MsRNA-nef, blue triangles) and Extra (carrying virion-enclosed HIV-1 RNA, chocolate squares). Thick lines and symbols represent the geometric means of the five patients from the study by Fischer et al. [12]. Thin transparent lines for each color represent the original data for each subclass of cells of each individual patient. The dashed line represents the limit of detection that was set at 50% of the lowest measured cell count. Measurements below this threshold were assumed to be at 50% of the detection limit to include them in the mean.

We first devised a detailed model of the within-host dynamics of HIV-1 that is based on the observations of different subclasses of HIV-1-infected cells in the study by Fischer et al. [12]. The five subclasses are HIV-1 DNA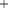, low, medium and high HIV-1 RNA expressing and cells that have virion-enclosed HIV-1 RNA associated with them (also see *Methods*). These subclasses show distinct decay dynamics in patients on cART (Figure 2). The slow decay of the subclass of PBMCs that contains proviral DNA (*DNA* 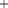) indicates that this cell population primarily contributes to the third phase decay and likely consists of defectively or latently infected cells to a large extent. The subclass of cells exhibiting UsRNA only (*Low*) decays slowly and most likely consists mainly of latently infected cells with low basal transcription of HIV-1. The cells with medium transcriptional activity (*Mid*) appear to contribute to the second and the third phase viral decay, which is characteristic of persistently and latently infected cells. The early drop in PBMCs with a higher transcriptional activity (*High*), which is more pronounced compared to cells with a low and medium transcriptional activity, that is followed by a slower loss of cells is reminiscent of activated, virus-producing and persistently infected cells. Finally, the PBMCs that have extracellular virion-enclosed HIV-1 RNA associated with them (*Extra*) show a very rapid loss before reaching the limit of detection. This is expected as they should represent the short-lived population of virus-producing cells [4] that contribute to the first phase of viral decay.

**Figure 3:**
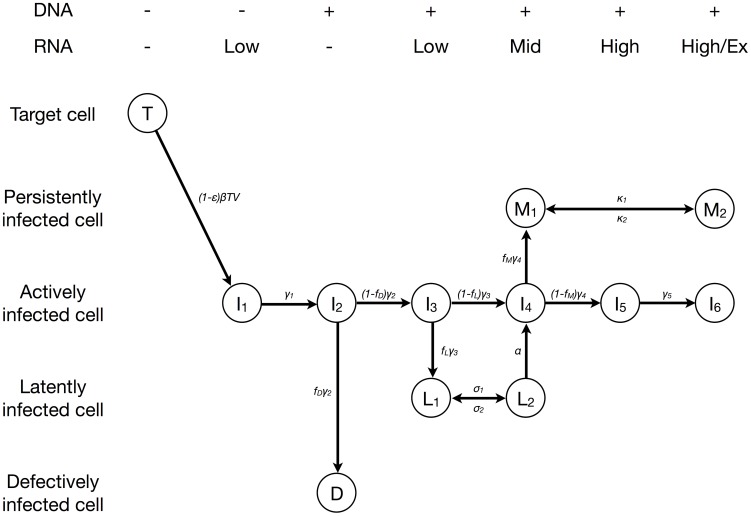
Model of HIV-1 dynamics. Actively infected cells move through an intracellular eclipse phase (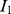 to 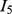) before they start to produce virus particles (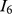). Some of the cells during the intracellular eclipse phase become either defectively infected (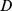), latently infected (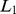) or persistently infected (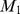). Both latently (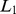 and 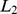) and persistently (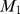 and 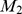) can move between two transcriptional states. Persistently infected cells that are in a high transcriptional state (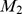) also contribute to virus production. The different subpopulations of infected cells can be stratified according to their HIV-1 DNA and RNA content (shown on top).

The different subclasses of HIV-1-infected cells clearly overlap and are representative of heterogeneous cell populations. Furthermore, the life cycle of HIV-1 from infection of a cell to the release of virus particles can be divided into cell populations with different transcriptional activity [27]. We took both of these important characteristics into account in our model that consists of 12 subpopulations of cells that can be stratified according to their HIV-1 DNA and RNA content (Figure 3 and *Methods*). In this model, we defined persistently infected cells (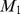 and 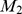) as long-lived cells that can produce viral particles. Latently infected cells (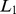 and 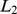) were assumed to transcribe HIV-1 RNA at low or intermediate levels [12], [13]. Infected cells that are HIV-1 DNA positive, but HIV-1 RNA negative, were assumed to remain transcriptionally silent during the observation period and considered as defectively infected cells (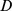).

**Figure 4:**
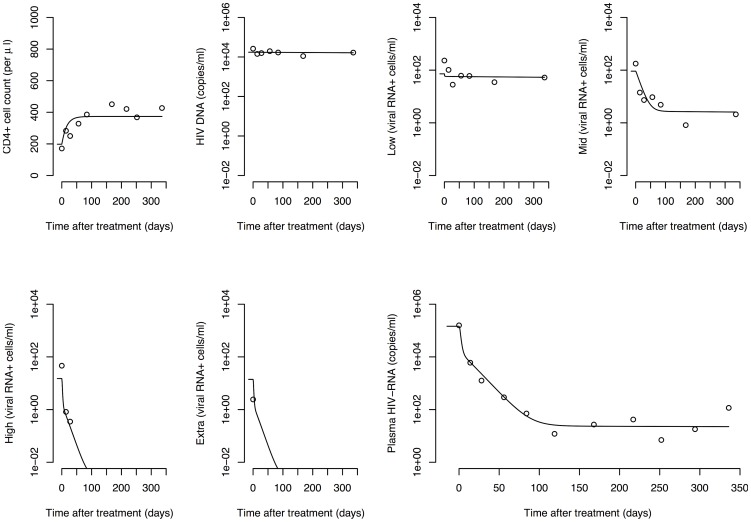
HIV-1 dynamics during cART. Circles denote measured data of patient 112 and lines represent the best fit of the default model. Model fits to data of the four other patients are given in *Text S1*.

**Figure 5:**
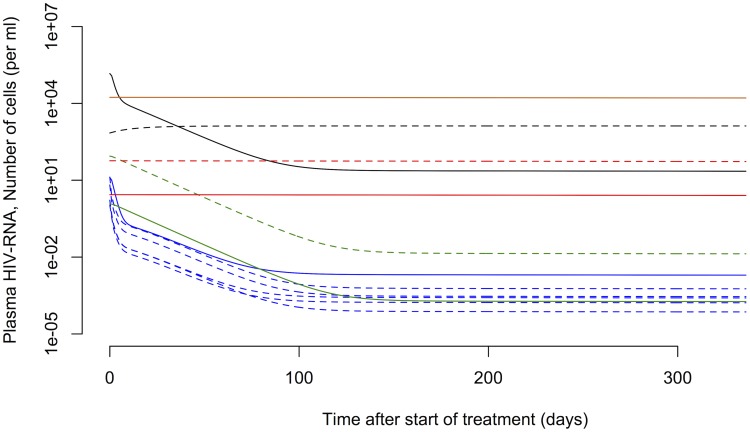
Dynamics of HIV-1-infected subpopulations during cART. The 12 different cellular subpopulations from Figure 3 (patient 112) are shown together with the virus. CD4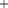 target cells (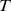): black dashed line; actively infected cells during the intracellular eclipse phase (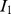 to 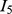): blue dashed lines; activated, virus-producing cells (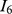): blue solid line; defectively infected cells (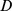): chocolate line; latently infected cells (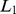 and 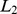): red dashed and solid line, respectively; persistently infected cells (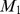 and 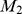): green dashed and solid line, respectively; virus particles (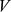): black solid line. The dynamics of the cellular subpopulations for all other patients are given in *Text S1*.

Fitting the mathematical model to the data from five HIV-1-infected patients resulted in a good description of the viral and cellular decay kinetics during cART (Figure 4 and *Text S1*). The individual dynamics of each subpopulation of cells are shown separately (Figure 5). The model clearly describes more pronounced decay dynamics in infected cells with increasing transcriptional activity. Table 1 provides a summary of the geometric means as well as the ranges of the best fit parameter estimates that describe the virus dynamics in each of the five patients. We found that 1.1% (0.2%–7.0%) of all CD4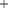 T cells can be target cells for infection with HIV-1. We also obtained estimates for the average lifespans of target cells (61 days, range: 11–528 days) and latently infected cells (33 years, range: 168 days–505 years). While others have estimated the average half-life of latently infected cells to be 6.3 months [28] and 44 months [29], our estimates are less precise due to the much shorter follow-up period after start of cART. However, the estimated activation rate of latently infected cells (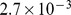 d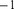, range: 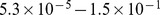 d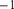) that also influences the slope of the third phase decay in plasma HIV-1 RNA is consistent with previous findings [30].

**Table 1:** Estimated parameters of HIV-1 dynamics.

Parameter	Explanation and reference	Constraint	Estimate (average)	Estimate (range)	Unit
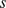	Proportion of CD4 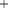 T cells that are target cells	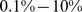	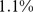	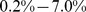	–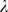	Production rate of target cells	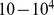	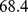	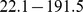	cells ml 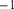 d 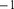
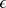	Drug effectiveness [23]	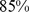	–	–	–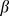	Infection rate	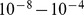	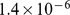	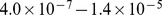	ml virion 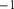 d 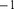
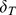	Death rate of target cells and other infected cells	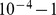	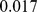	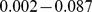	d 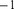
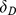	Death rate of defectively and latently infected cells ( 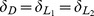 )	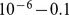	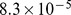	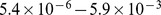	d 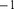
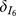	Death rate of virus-producing cells ( 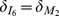 ) [72]	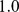	–	–	d 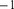
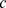	Clearance rate of free virus particles [17]	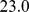	–	–	d 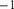
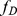	Fraction of 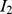 that become defectively infected cells	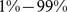	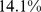	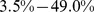	–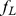	Fraction of 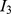 that become latently infected cells	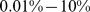	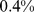	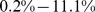	–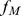	Fraction of 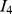 that become persistently infected cells	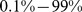	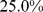	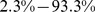	–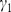	Transition rate from 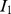 to 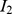 [27]	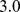	–	–	d 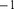
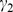	Transition rate from 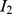 to 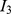 [27]	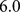	–	–	d 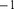
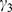	Transition rate from 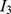 to 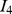 [27]	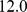	–	–	d 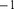
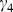	Transition rate from 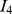 to 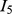 [27]	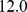	–	–	d 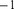
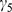	Transition rate from 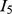 to 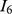 [27]	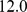	–	–	d 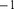
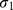	Transition rate from 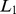 to 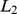	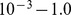	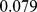	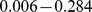	d 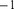
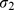	Transition rate from 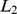 to 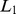 (assumption)	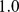	–	–	d 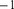
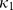	Transition rate from 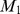 to 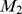	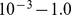	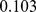	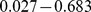	d 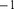
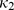	Transition rate from 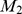 to 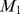 (assumption)	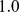	–	–	d 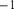
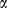	Activation rate of latently infected cells	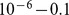	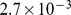	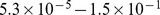	d 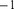
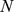	Viral burst size of virus-producing cells infected cells	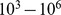	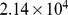	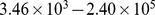	virions cell 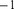

Estimates are given as geometric means including the range over all five patients. Parameters without an estimate (–) were assumed to be fixed during the fitting procedure. Intermediate values of the logarithmic range of constraint were used as starting values for the model fitting.

The parameters 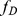, 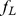 and 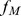 denote the fractions of cells that end up in a particular subpopulation in a sequential process during the intracellular eclipse phase. From this, we can calculate the average proportion of newly infected cells that become a certain cell type (*Text S1*). In contrast to another study [26], we find that only 63.4% (0.2%–7.0%) of infected cells become activated, virus-producing cells (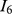). A substantial fraction of infected target cells results in defectively (14.0%) and persistently infected cells (21.2%). The proportion of infected cells that become latently infected or die before ending up in one of the subpopulations is small (0.3% and 1.1%, respectively). Note that after activation, latently infected cells can then either become persistently infected or activated, virus-producing cells by moving through cell class 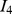. Transcriptional bursts that increase the level of viral RNA transcription occur on average every 12.7 days (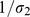, range: 3.5–165.2 days) and 9.7 days (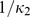, range: 1.5–37.0 days) in latently and persistently infected cells, assuming that bursts last for one day on average (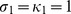 d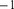). The total number of virus particles produced by a cell during its lifetime, the viral burst size, was estimated at 21′000 virions per cell (range: 3′500–240′000 virions per cell). Note that we assumed that persistently infected cells in an elevated transcriptional state (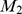) produce viral particles at the same rate as activated, virus-producing cells. However, the duration of virus release is shorter in persistently infected cells as they can revert to a lower transcriptional state (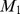). The majority of virus particles is produced by activated, virus-producing cells 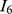 (68.3%, range: 5.6%–98.1%) with the remaining proportion being produced by persistently infected cells 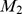. The high viral burst size suggests that the total number of virus-producing cells in peripheral blood must be small and we indeed found an average of only 25.7 cells ml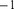 (range: 7.8–143.1 cells ml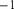) in the model during the chronic phase of infection.

**Figure 6:**
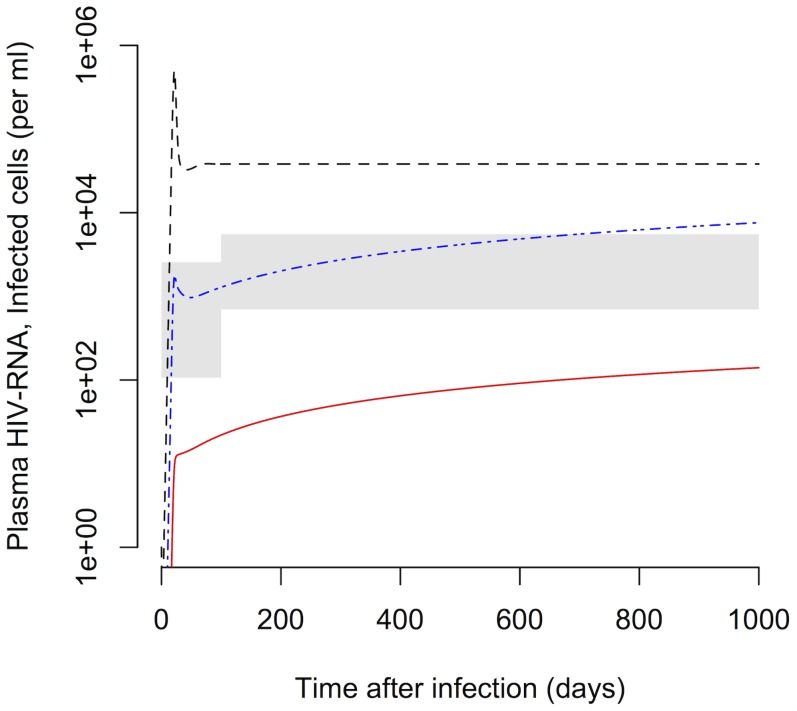
Development of the latently infected cell pool during acute and chronic of HIV-1 infection. The pool of latently infected cells (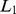
+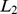) is shown as a red line, the number of HIV-1 DNA positive cells as a blue dash-dotted line and the plasma HIV-1 RNA as a black dashed line. The average parameter estimates from Table 1 were used for the model simulation. The gray areas represent two standard deviations around the mean of the number of HIV-1 DNA positive cells in patients that initiated cART during acute and chronic infection [33] (for details, see *Text S1*).

The parameters were estimated by fitting the virus dynamics model to data of patients chronically infected with HIV-1. Although there are mathematical models that describe acute and chronic HIV infection together [31], [32], the virus dynamics during acute infection could differ significantly due to different parameter values and even model structures. Nevertheless, our model can still be used to simulate the virus dynamics during the acute phase and compare the results to experimental and clinical data. We used the average of the estimated parameters to simulate early infection with HIV-1 from a small viral inoculum in a hypothetical patient. We set 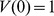 copy per ml and assumed that the target cells are at steady-state (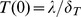). The rapid rise of plasma HIV-1 RNA during the first weeks of infection is followed by the chronic phase at which the virus concentration reaches its set-point level (Figure 6). The total pool of latently infected cells (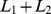) show somewhat different dynamics during acute HIV-1 infection. A very rapid expansion of latent cells during the viral growth phase is followed by a slower increase into the chronic phase of infection. From the time of peak viremia (22 days) to the chronic phase (1000 days), the latently infected cell pool expands 14.3-fold from 9.8 to 140.4 cells per ml. The expansion of the total number of HIV-1 DNA positive cells from the acute (1813 cells per ml) to the chronic phase (7608 cells per ml) is smaller (4.2-fold). This is consistent with the 3.8-fold difference in the number of HIV-1 DNA copies that were measured in patients that initiated cART during the acute and chronic phase from another study [33, and see *Text S1*]. The time after infection at which latently infected and HIV-1 DNA positive cells reach 50% of their chronic level is 441 and 451 days, respectively. Altogether, this illustrates the opportunity for eradication strategies during early cART interventions as the pool of HIV-1 infected cells seems to be substantially smaller during acute infection than during chronic infection.

## Discussion

We present the first mathematical model of virus dynamics that groups the different subpopulations of HIV-1-infected cells according to their transcriptional profile. The model assumes a heterogeneous population of latently and persistently infected cells having occasional transcriptional bursts to increase their level of RNA transcription which is consistent with experimental data from Fischer et al. [12]. Fitting this model to the unique data of virus transcription levels at the single cell level resulted in new estimates of the HIV-1 dynamics *in vivo*. We found that a large fraction of infected cells become either defectively or persistently infected cells. Furthermore, we found that the viral burst size can be high, between 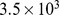 and 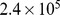 viral particles per virus-producing cell. Lastly, we simulated the acute phase of HIV-1 infection in a typical patient. This illustrated that the latently infected cell pool becomes rapidly established during the first months of acute infection and shows a slow increase during the first years of chronic infection.

Our study is unique in that we fit a mathematical model of HIV-1 within a host to data of the dynamics of different subclasses of infected cells. This is a substantial step beyond modeling studies that considered free virus in plasma, CD4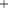 T cells and bulk measurements of viral activity only. The new quantitative insights into the replication dynamics of HIV-1 *in vivo* that this study provides will be useful for an improved understanding of HIV and the effects of novel treatment strategies.

The measurements of HIV-1-infected cells and the virus concentration were performed in blood only. In our mathematical model, we thus assume homogeneous mixing of virus and cells throughout the body. It is important to note, however, that the characteristic decay profile in the study by Fischer et al. [12] could also be a result of differential trafficking of virus particles and HIV-1-infected subpopulations of cells between the blood and lymphoid tissue. It has also been suggested that the virion clearance rate from the blood corresponds to a virion efflux to other organs where the virus is ultimately cleared [34]. Furthermore, non-productively infected CD4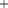 T cells could also indirectly die through ‘bystander’ effects [35], [36]. Finally, the typical second-phase decay could also result from virus production in infected macrophages [20] or heterogeneity in activation rates of latently infected cells [30], [37].

The concept of persistently infected cells has been previously used in mathematical models of HIV-1 dynamics to describe a population of long-lived cells that can contribute to the second-phase decay of virus during cART [20], [26]. Since the cellular subclass with medium transcriptional activity (*Mid*) seems to be rather long-lived and strongly characterized by a decay dynamics that could contribute to the second-phase decay of virus, we assumed that the majority of persistently infected cells belong to this class. This is consistent with the notion that persistently infected cells could be in a lower state of activation [21]. The contribution of other subpopulations of cells to the subclass *Mid* is small as the average lifespan of those cells is longer (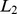) or shorter (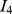) than that of persistently infected cells. It remains to be determined whether persistently infected cells could indeed release viral particles as a result of an increase in their transcriptional level. However, the reversion of virus-producing cells into a lower state of activation has been proposed previously [30]. The data did not allow estimation of both the frequency and duration of transcriptional bursts that lead to the release of virions in persistently infected cells. We assumed that once persistently infected cells release viral particles, the probability to die through cell lysis is the same as the probability of reversion.

For simplicity, we considered only one type of CD4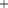 target cell whereas HIV-1 can infect activated but also resting CD4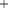 T cells. Our estimate of the proportion of CD4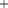 T cells that are target cells (1.1%) is somewhat lower than the 6.5% of CD4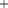 Ki-67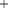 T cells in HIV-1-infected individuals that have been measured previously [38]. Also, the estimated average lifespan of target cells was longer than what others have estimated for activated cells [39]. The target cells in the model thus represent a particular subset of CD4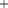 T cells that is smaller than the population of activated cells but has a longer average lifespan. The longer lifespan of target cells results from the assumption that the death rates of cells during the intracellular eclipse phase (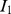 to 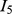) and persistently infected cells (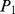) remain the same after infection, i.e., are the same as the death rate of uninfected target cells (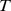). While persistently infected cells are indeed defined as long-lived cells that can produce virus, some studies have suggested that infected cells in the eclipse phase could also be a target of cytotoxic T lymphocyte (CTL) killing and experience high death rates [25], [40]–[42]. The early steps of proviral transcription also remain elusive. It has been suggested that the decay of non-integrated viral DNA in infected cells could render them CD4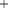 target cells again [43]–[46]. The kinetics of HIV-1 DNA indeed show a small drop early after start of cART (Figure 2 and ref. [47]). However, we have excluded this effect for simplicity. Ultimately, the mechanisms of viral latency in HIV-1 remain a matter of debate [48].

In our model, we assumed that after proviral insertion some cells fail to increase viral RNA transcription and become latently infected cells. Latency could also result from infection of resting CD4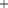 T cells or de-activation of activated CD4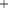 T cells. We have not included the latter two mechanisms in our model as the data would not allow us to distinguish between them.

The complexity of the HIV-1 life cycle and its mathematical representation prevents the identification of a ‘true’ underlying model. We made several simplifying assumptions in our default model but we also studied a series of alternative models and found that some of those models also fit the data well (Table S1 in *Text S1*). Importantly, the estimates of critical parameters such as the viral burst size, the proportion of CD4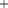 T cells that are target cells, and the fractions of cells that become defectively, latently or persistently infected in the alternative models that fit the data well were very similar to those estimated with the default model (Table S2 in *Text S1*).

We were also able to reject some competing hypotheses about the life cycle of HIV-1 (Table S1 in *Text S1*). Removing the intracellular eclipse phase, that contains infected cells at different stages with increasing levels of viral transcription, impairs the model fit. Assuming that latently or persistently infected cells are homogeneous subpopulations results in a substantially worse fit to the data. The limited number of data points and patients prevented a more thorough analysis and resulted in substantial uncertainty in estimating the model parameters. The wide ranges of estimates in Table 1 illustrate that the reported parameter values need to be treated with caution. We also used the least-squares method to fit the model to the data and did not consider maximum likelihood approaches [49], values below the limit of detection or nonlinear mixed-effect models [50].

It remains to be determined how well the parameter estimates that were obtained during the chronic phase of infection represent the situation of acute HIV-1 infection. It is re-assuring that the simulated virus dynamics of acute infection show a peak around three weeks after infection, which is in agreement with observations in patients [51], [52]. Nevertheless, differences in immune activation during acute infection are likely to result in different proportions of cells becoming latent upon infection and different activation rates of latently infected cells. Hence, our results on the development of the latently infected cell pool during acute infection need to be interpreted with caution.

We found the HIV-1 burst size *in vivo* to be large, corroborating previous estimates from Chen et al. [53] who found the average burst size in SIV-infected rhesus macaques to be between 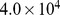 and 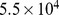. This is higher than other estimates that were in the range of 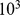 virions per cell [54], [55] and suggests that the number of virus-producing cells must be lower than previously anticipated. Measurements of extracellular virion-enclosed HIV-1 RNA (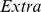) in the study by Fischer et al. [12] suggest that the number of productively infected cells in peripheral blood is small which is also reflected in our model fits. In contrast to other studies that assumed the viral production rate in long-lived persistently infected cells to be lower than in activated, virus-producing cells [56], we considered the viral production rates to be the same in both cell types. However, in our model persistently infected cells can have occasional transcriptional bursts from 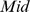 to 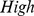, where they can release virus particles before reverting back to a lower transcriptional state or dying.

Our simulations of the development of different pools of HIV-1-infected cells are in good agreement with observations in patients. We find that the total number of HIV-1 DNA positive cells rapidly build up during the acute stage of infection. A very similar expansion was found in a recent study that measured the total number of HIV proviruses in PBMCs during the first weeks of HIV infection [57]. Also, our predicted ratio of the number of HIV-1 DNA positive cells during acute and chronic infection is in the same range as previously reported [33], [47]. The study by Murray et al. [47] further suggested that the level of HIV DNA continuously increases with duration of infection, reaching its 50% level at two years after infection. This contradicts earlier findings of stable levels of HIV-1 DNA positive PBMCs during the natural course of infection [58]. Our model predicts that the number of HIV-1 DNA positive PBMCs increases slowly during the first years of chronic infection and reaches its 50% level at 451 days after infection, corroborating the findings by Murray et al. [47].

An important question that remains is how many of HIV-1 DNA positive cells are latently or defectively infected. We found that the fraction of cells becoming defectively infected is surprisingly high. On the one hand, this could be a result of the assumption that HIV-1 DNA positive cells without viral RNA transcription remain silent. Some of these cells could actually be activated and start to produce UsRNA at low levels, i.e., become cells of the latent class 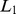. Eriksson et al. [59] measured a 300-fold difference between the number of latently infected cells as measured with a viral outgrowth assay and the total number of HIV-1 DNA positive resting CD4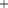 T cells. However, Ho et al. [60] showed a substantial fraction of noninduced proviruses in cells that have been stimulated in a viral outgrowth assay are replication-competent. They found that that the frequency of intact noninduced proviruses was at least 60-fold higher than the frequency of proviruses induced in a viral outgrowth assay. The median frequency of cells with intact non–induced proviruses per HIV-1 DNA positive resting CD4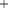 T cells was estimated at 3.7% [60]. In our simulation, the fraction of latently infected cells (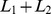) in all HIV-1 DNA positive cells (*DNA* 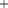) is 1.8% (140.4/7608) during chronic infection. The striking correspondence of these numbers suggests that our mathematical model realistically describes the dynamics of the latent reservoir. Since the subpopulation of 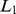 is much larger than 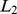, the majority of latently infected cells consist of PBMCs that contain solely HIV-1 UsRNA (*Low*), indicating that this transcriptional subclass is a good marker for viral latency.

This study provides an important step towards a more quantitative understanding of the dynamics of HIV-1 *in vivo*, in particular of the generation and maintenance of latently infected cells. A better understanding of the number of latently infected cells during acute infection is crucial for evaluating and predicting the outcome of early treatment and eradication strategies. Early cART treatment has been suggested to facilitate long-term control of HIV-1 [61] and studies have shown that it results in lower viral load levels during chronic infection [62]. Although the effects on viral load might only be transient [63], early treatment can prevent the expansion of viral cellular reservoirs in peripheral blood [33]. More recent strategies aim towards depletion of this reservoir [9], preferably during acute infection [64]. Predicting the chances of such eradication strategies critically depends on the ability to accurately quantify the pool of latently infected cells at various time points during HIV-1 infection. Our study supports the experimental finding that the latent reservoir becomes rapidly established during the first months of infection, and shows that the reservoir represents a significant proportion (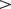
1%) of all HIV-1 DNA positive PBMCs during chronic infection. In addition, our mathematical model realistically describes the dynamics of different HIV-1-infected subpopulations of cells which will be useful for projecting the effects of eradication strategies.

## Materials and Methods

### Patient data

We used previously published data from five chronically HIV-1-infected therapy naive patients that initiated cART using reverse transcriptase and protease inhibitors (patient numbers: 103, 104, 110, 111, 112) [12]. Plasma HIV-1 RNA (copies per ml) and CD4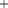 T cells (per µl) were measured at several time points during the first 48 weeks of cART. PBMCs were purified at weeks 0, 2, 4, 8, 12, 24 and 48 after the start of cART as described in Fischer et al. [65]. Serial dilution of PBMCs and patient matched PCR quantification of HIV-1 RNA species and DNA was performed as described elsewhere in detail [12], [13], [66], [67]. The freeze-thaw nuclease digestion method to differentiate between intracellular and virion encapsidated HIV-1 RNA has also been previously described in detail [4], [33]. HIV-1 RNA or DNA positive cell fractions measured as cells per 10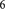 PBMCs were converted to number of cells per ml of blood by multiplying with the number of PBMCs per ml. This ultimately lead to the stratification of cells to five (partially overlapping) subclasses [12]:

- *DNA* 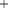: PBMCs containing HIV-1 DNA
- *Low*: PBMCs containing solely HIV-1 UsRNA
- *Mid*: PBMCs containing only HIV-1 MsRNA-tatrev or MsRNA-nef
- *High*: PBMCs containing elevated levels of both HIV-1 MsRNA-tatrev and MsRNA-nef
- *Extra*: PBMCs carrying virion-enclosed HIV-1 RNA

For the subclass *DNA* 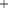, we make the assumption that there is only one proviral DNA copy per infected cell [68].

### Mathematical model

We devised a new virus dynamics model (Figure 3) which is adapted from previously published models [19], [25], [26], [30]. The various subpopulations of infected cells were stratified according to their HIV-1 DNA and RNA content. The model can be described by the following set of ordinary differential equations (ODEs): 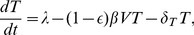
(1)

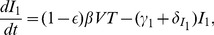
(2)

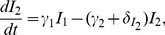
(3)

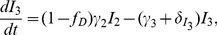
(4)

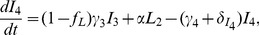
(5)

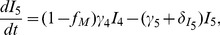
(6)

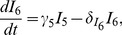
(7)

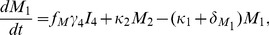
(8)

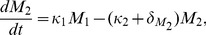
(9)

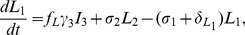
(10)

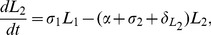
(11)

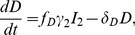
(12)

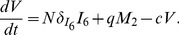
(13)


CD4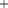 target cells, 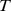, are produced at rate 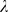 and can become infected by virus particles, 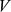, at rate 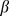. 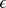 denotes treatment efficacy, where 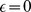 before the start of antiretroviral therapy. Newly infected cells move through the intracellular eclipse phase, where 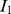 denotes the stage of reverse transcription, 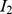 the stage of proviral integration, and 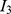 to 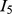 subsequent stages with increasing transcriptional activity. After the intracellular eclipse phase, activated, virus-producing cells, 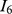, start to release free virus particles with a total viral burst size 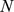. Some of the cells during the intracellular eclipse phase can become defectively infected cells, 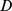, latently infected cells, 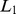, or persistently infected cells, 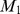. While we assume that defectively infected cells remain transcriptionally silent, both latently and persistently infected cells can exhibit transcriptional bursts that rise their transcriptional profile from *Low* to *Mid* and *Mid* to *High*, respectively. Latently infected cells in an elevated transcriptional state 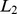 can become activated at rate 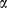 or move back to the lower transcriptional state 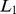. Similarly, persistently infected cells that are highly transcriptionally active 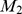 can release free virus particles at rate 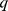 before they revert to a state of lower transcriptional activity or die. 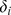 and 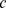 describe cell death and viral clearance rates, respectively.

Due to the complexity of the full model, we make a number of simplifying assumptions. First, we assumed several of the cell death rates to be the same: the death rates of virus-producing cells 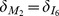 and the death rates of defectively and latently infected cells 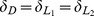. The death rates of infected cells that are not virus-producing and do not solely belong to a resting phenotype, such as defectively and latently infected, were kept the same as the death rate of target cells (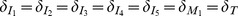). Second, the viral production rates in both virus-producing cells (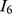 and 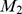) are kept the same, i.e., 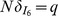. Note, however, that persistently infected cells (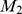) have a lower burst size than activated, virus-producing cells (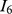) because they can revert to a non-productive state (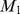). The default model described above is compared to a number of alternative models with different assumptions of the viral life cycle (*Text S1*).

### Model fitting

The default model contains 22 parameters of which 10 are fixed to previously used values from the literature or based on assumptions (Table 1). The remaining 12 parameters were constrained based on literature values and consensus and we used the geometric mean of the restricted range as starting values when fitting the model to data. This proved to be a good strategy for estimating the model parameters. The set of ODEs were solved numerically in the R software environment for statistical computing [69] using the function *ode* from the package *deSolve* [70]. The 12 model variables were initiated with the target cells at their steady-state (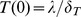), 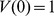 copy per ml, and all other variables being zero. We assumed that the chronic state of infection is reached after 1000 days (about three years), set 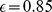 [23] and further integrated the system during the time on cART (336 days).

The concentration of free virus 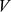 was measured directly but several of the infected cell populations contribute to the different subclasses of PBMCs (Figure 3): 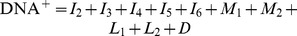, 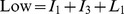, 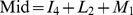, 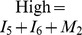 and 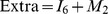. We further assume that target cells, 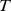, correspond to a fraction, 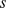, of all CD4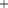 T cells. All 12 parameters (11 model parameters and one scaling parameter) were estimated by fitting the model to the data of each patient individually and minimizing the sum of squared residuals (SSR) between the prediction of the model and the data (taking the natural logarithm). All data points were weighted equally. However, the higher number of data points for free virus compared to cellular subclasses (e.g., 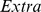) forced the model to fit the virus concentration better than the other variables. We used the minimization algorithm by Nelder & Mead [71] that is implemented in the function *optim* and the *parallel* package for parallel computation. The algorithm by Nelder & Mead is very robust in finding local optima. As a sensitivity analysis, we used different starting values for the parameters and the method *SANN* that is a variant of simulated annealing. Simulated annealing usually performs better in finding global optima but is relatively slow. In both cases, we found the best-fit parameter estimates to be the same or very similar to our default fitting strategy. Parameter estimates are presented as geometric means including the ranges over all five patients. Code files can be obtained freely upon request from the corresponding author.

## Supporting Information

Text S1 This file contains the calculation of the proportion of cell types, the definition and results of the alternative models, the fits of the default model to the data from the four other patients, and the calculation of HIV-1 DNA positive cells from the study by Schmid et al. [33].
(PDF)
