# Supplementary material for: Quantifying the Turnover of Transcriptional Subclasses of HIV-1-Infected Cells
Source: PLoS Comput Biol. 2014 Oct 23;10(10):e1003871. doi: 10.1371/journal.pcbi.1003871 (PMC4207463; doi:10.1371/journal.pcbi.1003871)
Supplement: Text S1 — This file contains the calculation of the proportion of cell types, the definition and results of the alternative models, the fits of the default model to the data from the four other patients, and the calculation of HIV-1 DNA positive cells from the study by Schmid et al. [33]. (PDF) [file pcbi.1003871.s001.pdf]

Quantifying the turnover of transcriptional subclasses of HIV-1-infected cells

Supporting Information - Text S1

Christian L. Althaus, Beda Joos, Alan S. Perelson, Huldrych F. Günthard

Contents

1 Calculation of the proportion of cell types 1

2 Alternative models 1

3 Model fits to data from other patients 5

4 HIV-1 DNA positive cells 10

1 Calculation of the proportion of cell types

After infection of target cells, infected cells I_1 can either become defectively infected (D), latently infected (L_1), persistently infected (M_1) or activated virus-producing cells (I_6) or die during the intracellular eclipse phase. The proportions of cells that end up in a particular subpopulation were calculated as follows:

$$\pi_D = \frac{\gamma_1}{\delta_{I_1}+\gamma_1} \times \frac{f_D\gamma_2}{\delta_{I_2}+\gamma_2}, \quad (1)$$

$$\pi_L = \frac{\gamma_1}{\delta_{I_1}+\gamma_1} \times \frac{(1-f_D)\gamma_2}{\delta_{I_2}+\gamma_2} \times \frac{f_L\gamma_3}{\delta_{I_3}+\gamma_3}, \quad (2)$$

$$\pi_M = \frac{\gamma_1}{\delta_{I_1}+\gamma_1} \times \frac{(1-f_D)\gamma_2}{\delta_{I_2}+\gamma_2} \times \frac{(1-f_L)\gamma_3}{\delta_{I_3}+\gamma_3} \times \frac{f_M\gamma_4}{\delta_{I_4}+\gamma_4}, \quad (3)$$

$$\pi_I = \frac{\gamma_1}{\delta_{I_1}+\gamma_1} \times \frac{(1-f_D)\gamma_2}{\delta_{I_2}+\gamma_2} \times \frac{(1-f_L)\gamma_3}{\delta_{I_3}+\gamma_3} \times \frac{(1-f_M)\gamma_4}{\delta_{I_4}+\gamma_4} \times \frac{\gamma_5}{\delta_{I_5}+\gamma_5}, \quad (4)$$

$$\pi_\delta = 1-(\pi_D+\pi_L+\pi_M+\pi_I), \quad (5)$$

where π_D, π_L, π_M, π_I and π_δ are the proportions of cells that become defectively infected, latently infected, persistently infected or activated virus-producing cells or die, respectively.

2 Alternative models

The results from the main text were based on our default model but we also constructed 12 alternative models (Table S1) that differ in one or more characteristics of the HIV-1 life cycle:

- **No CD4$^+$:** We did not include the number of CD4$^+$ T cells per μl in the model fits.

- $\boldsymbol{\delta_{I_6} = 0.5}$**:** The average lifespan of virus-producing cells (I_6 and M_2) is longer ($1/\delta_{I_6}$ = 2 days).

- $\boldsymbol{\epsilon = 1.0}$**:** Drug efficacy is assumed to be 100%.

- **Direct:** The fate of an infected cell is determined directly after infection and not sequentially during the intracellular eclipse phase. This means that target cells (T) can become defectively infected (D), latently infected (L_1) and persistently infected (M_1) directly after infection and not sequentially during the intracellular eclipse phase.

- **No eclipse:** There is no intracellular eclipse phase, i.e., no stages I_1 to I_5.

- **Hom. 1:** Latently infected cells were assumed to be a homogeneous cell population (no L_2).

- **Hom. 2:** Persistently infected cells were assumed to be a homogeneous cell population (no M_1).

- **Prod./death 1:** Persistently infected cells (M_2) and activated, virus-producing cells (I_6) have a different viral burst size.

- **Prod./death 2:** Persistently infected cells have a different death rate ($\delta_{M_2} = \delta_T$) than activated, virus-producing cells (δ_{I_6}), but the same viral burst size N.

- **Prod./death 3:** Persistently infected cells (M_2) and activated, virus-producing cells (I_6) have a both a different viral burst size and death rate than activated, virus-producing cells.

- **Burst 1:** Transcriptional bursts in latently and persistently infected cells last for one hour on average.

- **Burst 2:** Transcriptional bursts in latently and persistently infected cells last for one week on average.

Table S1 provides an overview of all models together with the sum of squared residuals (SSR) from fitting them to the data of the five patients. The estimated parameters of the HIV-1 dynamics for all models are given in Table S2.

It can be seen that the default model provides the best fit to the data together with the model where the fate of an infected cell is determined directly after infection (*Direct*). The model that does not include the number of CD4$^+$ T cells (*No CD4$^+$*) results in an only slightly smaller SSR than the default model. Different assumptions about the death rate of virus-producing cells (δ_{I_6} = 0.5) and the drug efficacy (ϵ = 1.0) do not improve the quality of the fits. Assuming a different viral burst size for persistently and activated virus-producing cells (*Prod./death 1* and *Prod./death 1*) is also consistent with the data. However, the estimated viral burst sizes in persistently infected cells were higher than in activated, virus-producing cells. This seems to be a rather unrealistic scenario. A longer duration of transcriptional bursts results in a reasonably good model fit (*Burst 2*) whereas bursts with a duration of only one hour are not consistent with the data (*Burst 1*). All other models (*No eclipse, Hom. 1, Hom. 2, Prod./death 2* and *Prod./death 2*) fit the data substantially worse than the default model. Note that some models do not converge, indicating unrealistic assumptions about the HIV-1 life cycle and/or too many free parameters (overfitting).

Table S1. Comparison of the alternative models with the default model from the main text.

Model	$CD4^+$	Cell fate	Eclipse phase	Latent	Persistent	Production	Death	Burst	No. of parameters	Convergence[a]	SSR[b]
Default	Yes	Sequential	Yes	Het	Het	Same	Same	Day	12	Yes	160.7
No $CD4^+$	No	Sequential	Yes	Het	Het	Same	Same	Day	11	Yes	159.1[c]
$\delta_{I_6} = 0.5$	Yes	Sequential	Yes	Het	Het	Same	Same	Day	12	Yes	165.6
$\epsilon = 1.0$	Yes	Sequential	Yes	Het	Het	Same	Same	Day	12	No	161.7
Direct	Yes	Direct	Yes	Het	Het	Same	Same	Day	12	Yes	160.2
No eclipse	Yes	Direct	No	Het	Het	Same	Same	Day	12	No	176.3
Hom. 1	Yes	Sequential	Yes	Hom	Het	Same	Same	Day	11	No	221.3
Hom. 2	Yes	Sequential	Yes	Het	Hom	Same	Same	Day	11	No	274.2
Prod./death 1	Yes	Sequential	Yes	Het	Het	Different	Same	Day	13	No	157.6
Prod./death 2	Yes	Sequential	Yes	Het	Het	Same	Different	Day	12	No	197.6
Prod./death 3	Yes	Sequential	Yes	Het	Het	Different	Different	Day	13	No	167.3
Burst 1	Yes	Sequential	Yes	Het	Het	Same	Same	Hour	12	No	245.5
Burst 2	Yes	Sequential	Yes	Het	Het	Same	Same	Week	12	Yes	165.5

[a] No if the fitting algorithm failed to convergence for one or more patients

[b] Sum of squared residuals

[c] Note that the SSR for the *No $CD4^+$* model is smaller due to a lower number of data points

Table S2. Estimated parameters of HIV-1 dynamics for all models. Values are given as geometric means over all five patients. For dimensions, we refer to Table 1 from the main text.

**Model**	s	λ	β	δ_T	δ_D	f_D	f_L	f_M	σ_1	κ_1	α	N	q
Default	1.14e-02	6.84e+01	1.35e-06	1.65e-02	8.25e-05	1.41e-01	3.51e-03	2.50e-01	7.89e-02	1.03e-01	2.67e-03	2.14e+04	–
No $CD4^+$	–	7.76e+01	6.46e-07	1.39e-02	3.67e-05	1.58e-01	3.90e-03	1.89e-01	7.86e-02	1.02e-01	2.53e-03	2.54e+04	–
$\delta_{I_6} = 0.5$	8.44e-03	7.24e+01	1.07e-06	2.71e-02	6.72e-05	2.28e-01	7.15e-03	2.16e-01	7.27e-02	1.16e-01	3.26e-03	3.16e+04	–
$\epsilon = 1.0$	2.28e-02	1.01e+02	4.81e-07	7.39e-03	3.00e-04	1.05e-01	4.02e-03	1.74e-01	6.31e-02	8.64e-02	8.37e-03	2.48e+04	–
Direct	2.06e-02	6.98e+01	6.86e-07	9.32e-03	1.32e-04	1.35e-01	2.74e-03	1.68e-01	8.09e-02	1.70e-01	2.14e-03	2.06e+04	–
No eclipse	1.91e-02	5.46e+01	6.77e-07	6.75e-03	6.99e-05	1.65e-01	3.02e-03	7.51e-02	6.81e-02	1.33e-01	2.40e-03	2.07e+04	–
Hom. 1	1.61e-02	6.12e+01	2.38e-06	7.79e-03	5.60e-05	1.63e-01	4.41e-03	9.72e-02	–	5.25e-02	4.94e-04	7.98e+03	–
Hom. 2	6.73e-02	1.19e+02	3.42e-07	3.74e-03	4.14e-04	1.20e-01	1.29e-02	4.87e-03	1.47e-01	–	3.89e-02	6.08e+03	–
Prod./death 1	1.99e-02	1.59e+02	6.03e-07	2.63e-02	4.19e-05	1.12e-01	2.77e-03	5.22e-02	7.63e-02	8.62e-02	5.44e-03	1.59e+04	3.73e+04
Prod./death 2	9.51e-03	8.54e+01	2.46e-07	2.95e-02	1.44e-04	2.67e-01	6.13e-03	4.96e-02	1.80e-02	9.62e-02	5.73e-04	1.24e+05	–
Prod./death 3	7.94e-03	1.14e+02	1.23e-06	4.73e-02	1.15e-04	2.57e-01	7.53e-03	7.26e-02	4.80e-02	2.99e-02	2.60e-03	5.22e+04	4.49e+03
Burst 1	3.89e-02	9.06e+01	3.33e-07	6.40e-03	3.08e-04	1.36e-01	2.65e-03	3.43e-02	8.92e-02	3.08e-01	1.76e-03	1.77e+04	–
Burst 2	7.27e-03	9.98e+01	3.23e-06	4.62e-02	1.38e-03	1.60e-01	5.06e-03	2.17e-01	1.34e-02	6.68e-02	3.80e-03	1.65e+04	–

3 Model fits to data from other patients

The fits of the default model (see main text) to the data of the four additional patients (103, 104, 110 and 111) from Fischer et al. [1] are shown in Figure S1–S4. The dynamics of each subpopulation of cells for each patient are shown in Figure S5.

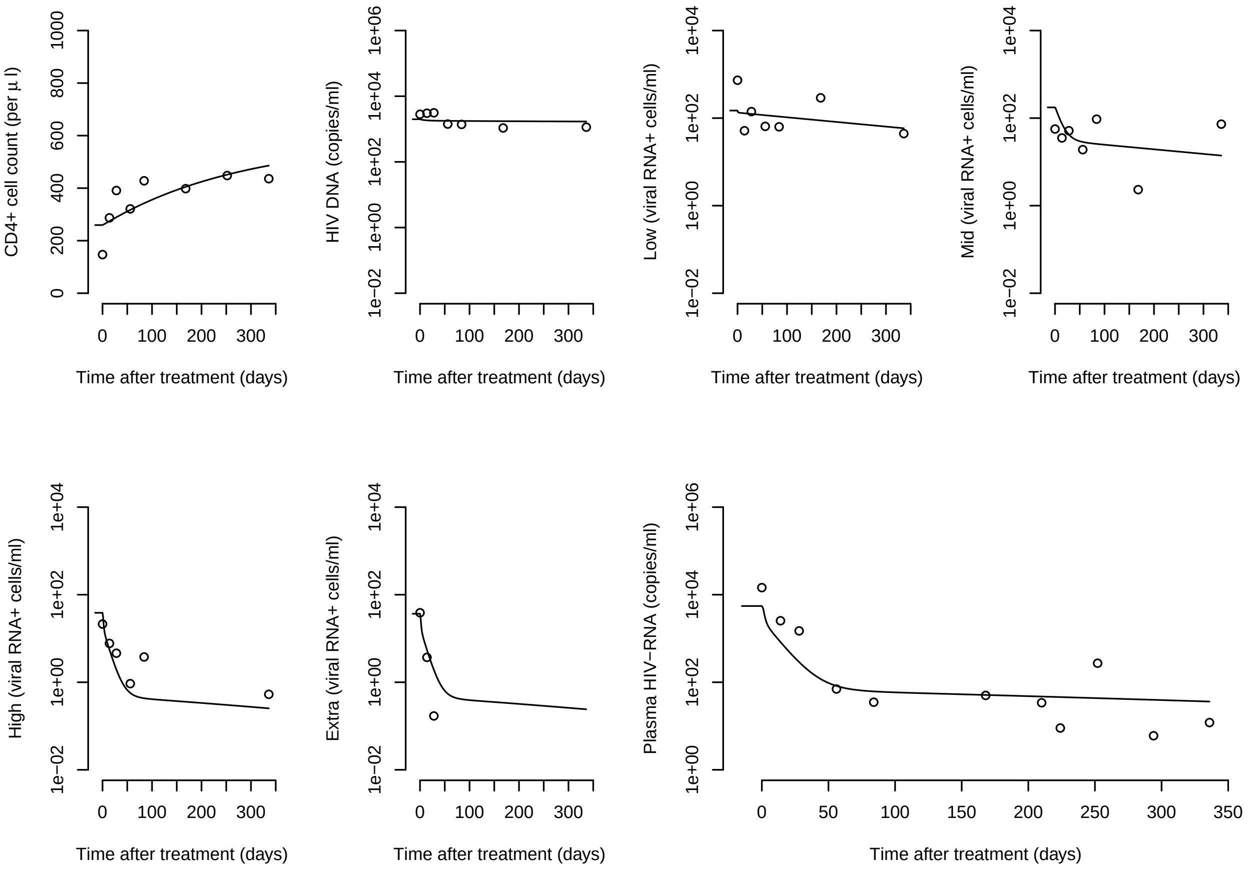

Figure S1. Model fit for patient 103. Circles denote measured data and lines represent the best model fit.

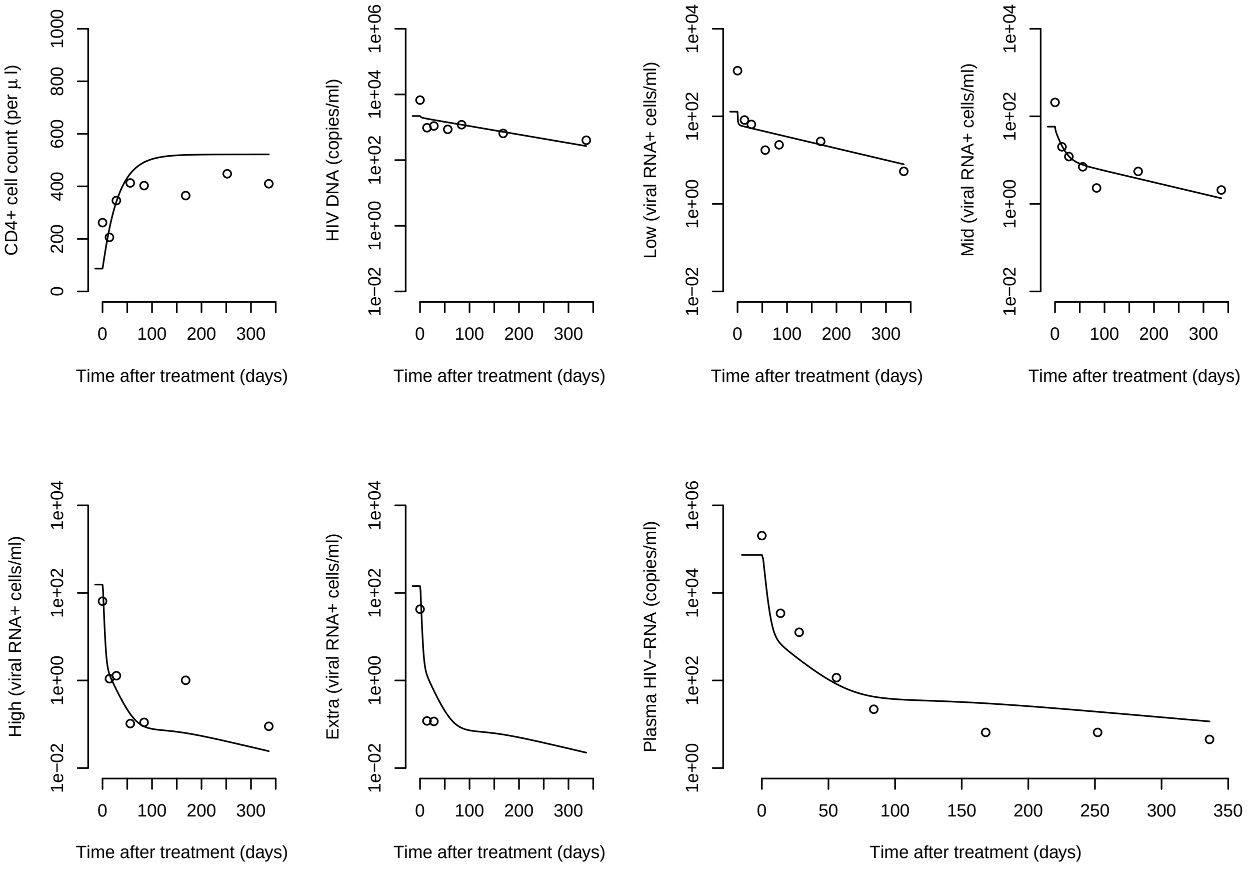

Figure S2. Model fit for patient 104. Circles denote measured data and lines represent the best model fit.

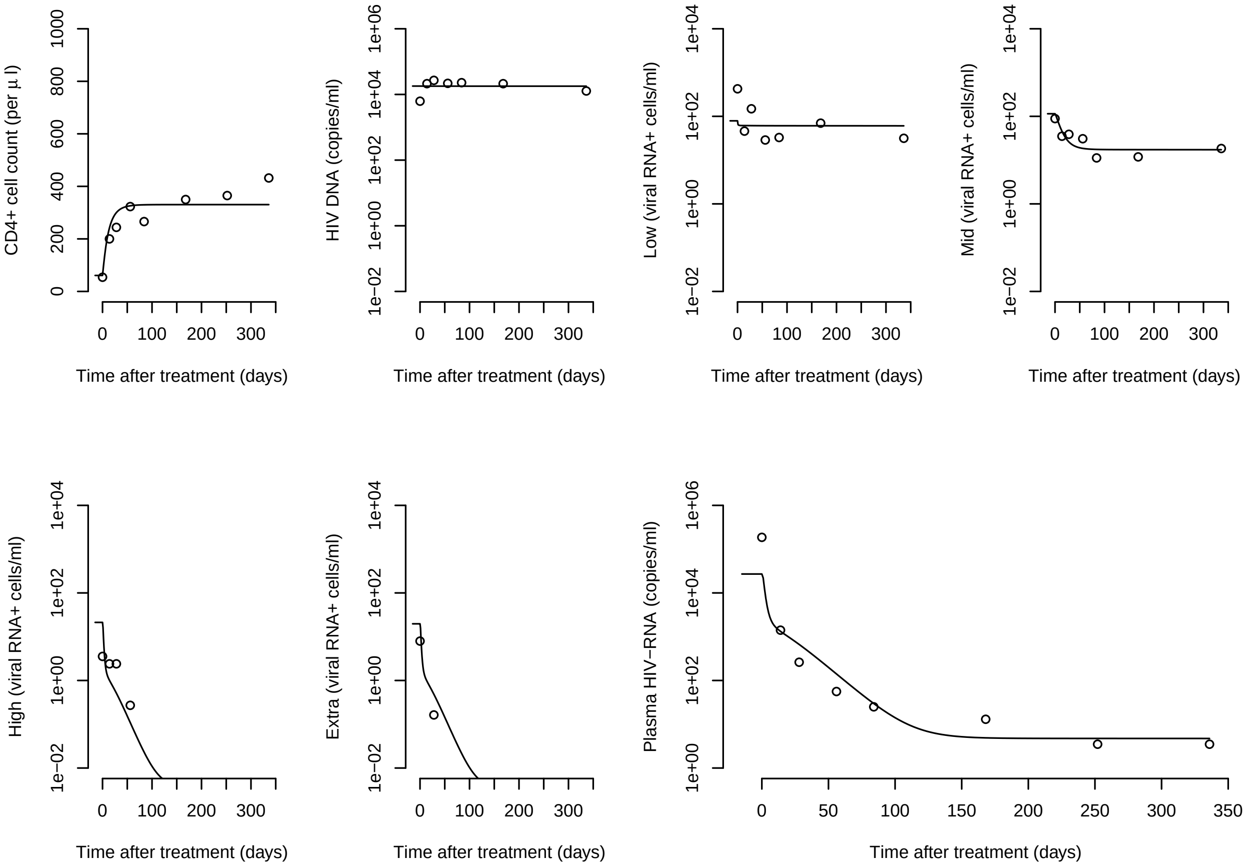

Figure S3. Model fit for patient 110. Circles denote measured data and lines represent the best model fit.

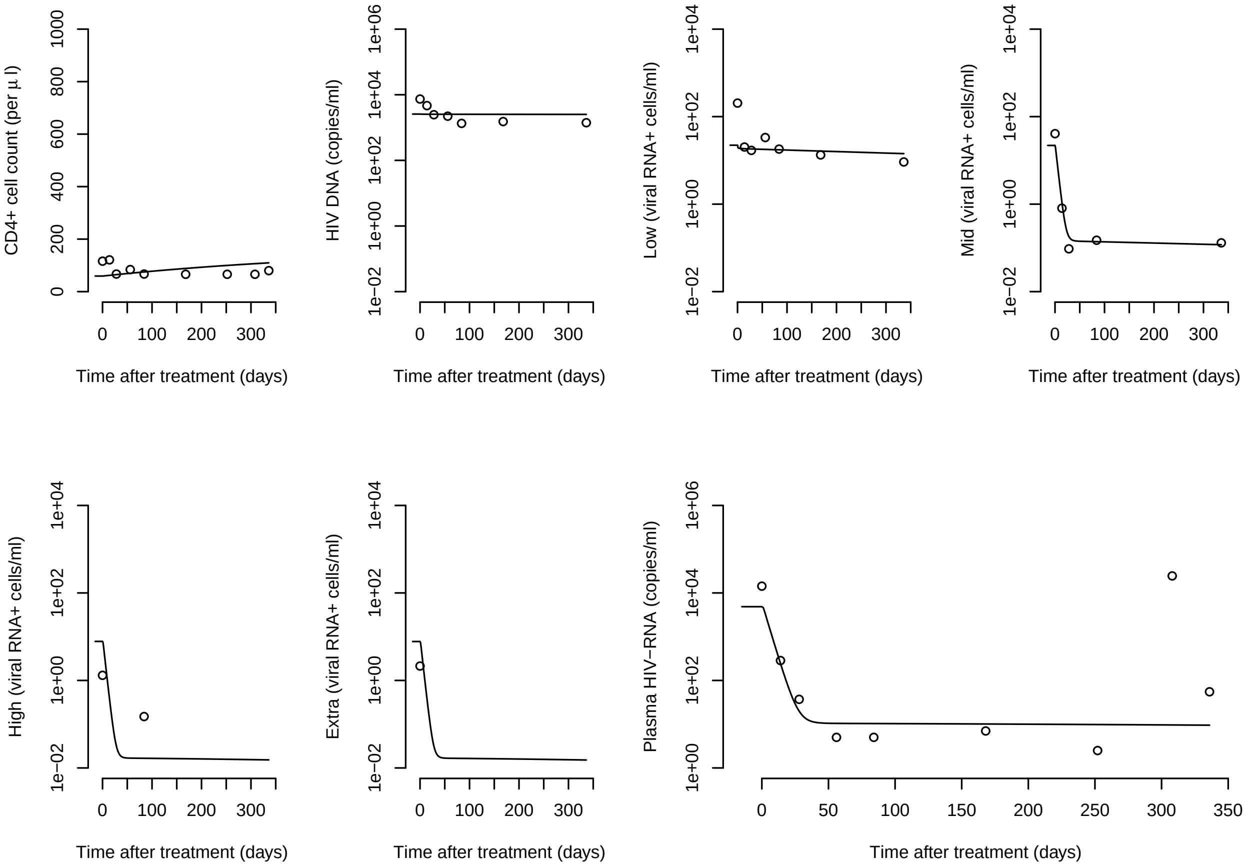

Figure S4. Model fit for patient 111. Circles denote measured data and lines represent the best model fit. We did not include the outlier in plasma HIV-RNA at 308 days after start of treatment in the model fit because treatment had to be interrupted due to side effects.

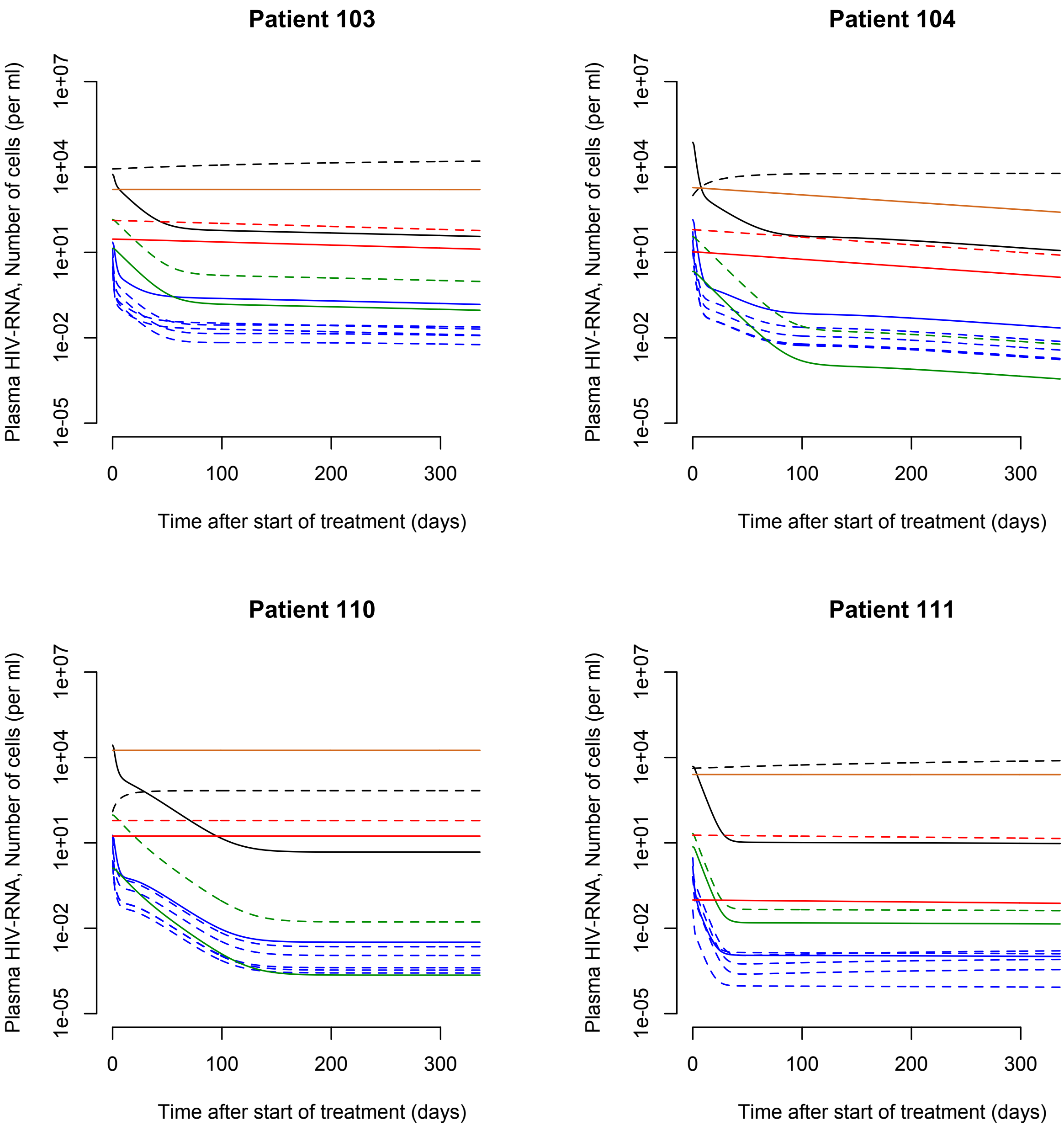

Figure S5. Dynamics of the cellular subpopulations for patients 103, 104, 110 and 111. CD4$^+$ target cells (T): black dashed line; actively infected cells during the intracellular eclipse phase (I_1 to I_5): blue dashed lines; activated, virus-producing cells (I_6): blue solid line; defectively infected cells (D): chocolate line; latently infected cells (L_1 and L_2): red dashed and solid line, respectively; persistently infected cells (M_1 and M_2): green dashed and solid line, respectively; virus particles (V): black solid line.

4 HIV-1 DNA positive cells

The study by Schmid et al. [2] measured the number of HIV-1 DNA copies per 10^6 peripheral blood mononuclear cells (PBMCs) in patients that initiated cART during the acute ($n = 24$) and chronic infection ($n = 15$). We assumed that there is only one proviral DNA copy per infected cell [3], and scale the number of HIV-1 DNA copies with the average number of PBMCs per ml from the five patients in the study by Fischer et al. [1]. This resulted in numbers of HIV-1 DNA positive cells per ml that are expected to exist during acute (mean: 522 cells) and chronic infection (mean: 1964 cells). Two standard deviations around the mean of those numbers are shown in Figure 4 from the main text.

References

1. Fischer M, Joos B, Niederöst B, Kaiser P, Hafner R, et al. (2008) Biphasic decay kinetics suggest progressive slowing in turnover of latently HIV-1 infected cells during antiretroviral therapy. Retrovirology 5: 107.

2. Schmid A, Gianella S, von Wyl V, Metzner KJ, Scherrer AU, et al. (2010) Profound depletion of HIV-1 transcription in patients initiating antiretroviral therapy during acute infection. PLoS One 5: e13310.

3. Josefsson L, King MS, Makitalo B, Brännström J, Shao W, et al. (2011) Majority of CD4+ T cells from peripheral blood of HIV-1-infected individuals contain only one HIV DNA molecule. Proc Natl Acad Sci U S A 108: 11199-204.
